# The Ventral Pallidum Innervates a Distinct Subset of Midbrain Dopamine Neurons

**DOI:** 10.1523/ENEURO.0222-25.2025

**Published:** 2025-10-23

**Authors:** Olivia J. Yang, Hannah B. Elam, Kayla Lilly, Alexandra M. McCoy, Valeriia Klepikova, Stephanie M. Perez, Daniel J. Lodge

**Affiliations:** ^1^Department of Pharmacology and Center for Biomedical Neuroscience, University of Texas Health Science Center, San Antonio, Texas 78229; ^2^South Texas Veterans Health Care System, Audie L. Murphy Division, San Antonio, Texas 78229

**Keywords:** dopamine, psychiatric disorders, schizophrenia, ventral pallidum

## Abstract

Aberrant dopamine transmission is a hallmark of several psychiatric disorders. Dopamine neurons in the ventral tegmental area (VTA) display distinct activity states that are regulated by discrete afferent inputs. For example, burst firing requires excitatory input from the mesopontine tegmentum, while dopamine neuron population activity, defined as the number of spontaneously active dopamine neurons, is thought to be dependent on inhibitory drive from the ventral pallidum (VP). Rodent models used to study psychiatric disorders, such as psychosis, consistently exhibit elevated dopamine neuron population activity, due to decreased tonic inhibition from the VP. However, it remains unclear whether the VP can modulate all dopamine neurons or if only a specific subset of VTA dopamine neurons receive innervation from the VP to be recruited as required. This knowledge is critical for understanding dopamine regulation in normal and pathological conditions. Here, we used in vivo electrophysiology in male and female rats to record VTA dopamine neurons inhibited by electrical stimulation of the VP. Specifically, VP stimulation inhibited ∼22% of spontaneously active dopamine neurons; however, activation of the ventral hippocampus, a modulator of VTA population activity, increased the proportion to ∼48%. This increase suggests that VP selectively modulates a subset of dopamine neurons that can be recruited by afferent activation. Anterograde monosynaptic tracing revealed that approximately half of the VTA dopamine neurons receive input from the VP. Taken together, we demonstrate that a subset of VTA dopamine neurons receives monosynaptic input from the VP, providing valuable information regarding the regulation of VTA neuron activity.

## Significance Statement

Dysregulated dopamine signaling has been linked to many psychiatric disorders. Therefore, understanding how dopamine neuron activity is regulated is essential for identifying mechanisms contributing to normal and pathologic states. Dopamine neuron population activity refers to a dynamic collection of neurons that can be recruited to assign salience to stimuli. This activity state is known to be regulated by afferent inputs to the ventral pallidum (VP), which provides a tonic inhibition to dopamine neurons. This study demonstrates that the VP provides flexible, targeted control of dopamine signaling. These findings improve our understanding of how dopamine activity is regulated and may help guide future treatments for psychiatric disorders involving dopamine dysfunction.

## Introduction

The dopamine system plays a central role in numerous processes, including motivation, reward learning, salience attribution, decision-making, and cognitive flexibility (Klein et al., 2019; Baik and Baik, 2020). When this system becomes dysregulated, it can lead to pathological states characterized by altered perception, affect, and motivation (Finlay and Zigmond, 1997; Klein et al., 2019; Baik and Baik, 2020). Indeed, aberrant dopamine signaling has been implicated in a wide range of psychiatric disorders, including schizophrenia, psychosis, depression, bipolar disorder, and substance use disorders (Grace, 1995, 2000; Volkow et al., 2004; Howes et al., 2009; Egerton et al., 2013; Belujon and Grace, 2014; Ashok et al., 2017; Compean and Hamner, 2019). In the case of schizophrenia and psychosis, patients exhibit a hyperdopaminergic state, particularly within subcortical regions such as the striatum (Abi-Dargham et al., 1998; Laruelle and Abi-Dargham, 1999; Howes et al., 2009, 2012). This increase in dopamine system function is thought to underlie the phenomenon of aberrant salience, where neutral or irrelevant stimuli are assigned inappropriate significance, contributing to the development of delusions and hallucinations (Kapur, 2003; Palaniyappan and Liddle, 2012). Thus, understanding the mechanisms that regulate dopamine system output is critical for elucidating the neural basis of psychiatric illnesses and identifying targets for therapeutic intervention.

**Figure 1.**
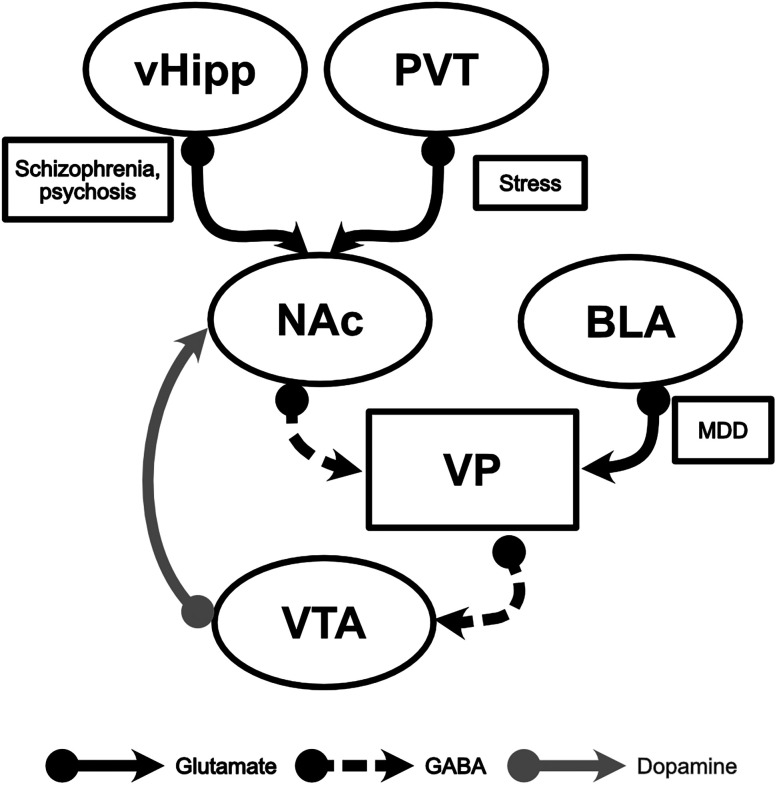
Schematic depicting afferent inputs to VP that modulate VTA dopamine neuron population activity and the role of these inputs in various pathological states. Ventral hippocampus (vHipp); paraventricular nucleus of the thalamus (PVT); nucleus accumbens (NAc); basolateral amygdala (BLA); ventral pallidum (VP); ventral tegmental area (VTA); major depressive disorder (MDD).

The cell bodies of mesolimbic dopamine neurons reside in the ventral tegmental area (VTA), and these neurons can exist in two distinct activity states: active (single spike and burst firing) and inactive (hyperpolarized; Grace and Bunney, 1984a,b). Advances in electrophysiological techniques have demonstrated that under normal conditions, only a certain number of dopamine neurons in the VTA are spontaneously active, while the rest are kept in an inactive, hyperpolarized state (Grace and Bunney, 1985). This observation led to the establishment of a third activity state, population activity, used to describe the number of spontaneously firing dopamine neurons. These “silent” dopamine neurons can be recruited to assign salience to an event or chronically altered under certain pathological states (Lodge and Grace, 2006). For example, rodent models used to study psychosis consistently demonstrated dramatic increases in dopamine neuron population activity (Lodge and Grace, 2007, 2009; Aguilar et al., 2014; Elam et al., 2021), while models used to study stress-induced depression-like behaviors report pathological decreases in activity (Chang and Grace, 2013a,b). Moreover, it has been previously shown that increased dopamine neuron population activity leads to increased dopamine synthesis capacity that is observed in rodent models and patients with psychosis (Howes et al., 2009, 2012; Perez et al., 2022). This subset of dopamine neurons is essential for regulating salience and the aberrant dopaminergic signaling seen in psychiatric disorders such as psychosis and depression. However, the mechanisms involved in this regulation are not yet fully understood. The regulation of dopamine neuron population activity is a complex process involving multiple brain regions (Fig. 1). Previous research showed that the ventral pallidum (VP) exerts a significant inhibitory influence on dopamine neurons through GABAergic projections to the VTA (Grace and Bunney, 1985; Floresco et al., 2001, 2003; Watabe-Uchida et al., 2012; Mahler et al., 2014). Glutamatergic inputs from the ventral hippocampus (vHipp) and paraventricular nucleus of the thalamus converge in the nucleus accumbens (NAc) to modulate projections to the VP (Perez and Lodge, 2018). Disruptions in the regulation of this circuit lead to the disinhibition of dopamine neurons through increased inhibitory drive from the NAc to the VP (Aguilar et al., 2014). Interestingly, rodent models used to study schizophrenia display increased dopamine neuron population activity due to heightened hippocampal activity, while rodents exposed to acute stressors, such as foot shocks, exhibit a similar phenotype, though this is mediated by hyperactivity in the thalamus (Floresco et al., 2001, 2003; Lodge and Grace, 2007, 2008b; Perez and Lodge, 2018; Elam et al., 2025). Conversely, decreases in dopamine neuron population activity are observed following chronic mild stress and are associated with a more depressive-like phenotype via increased excitatory transmission from the basolateral amygdala (BLA) to VP (Chang and Grace, 2013a,b). Although we have gained some insights, we still do not fully understand how the projections from the VP to VTA influence dopamine neuron activity. One possibility is that the VP forms synaptic connections with the majority of VTA dopamine neurons but selectively inhibits them based on specific inputs. Alternatively, the VP may target only a subpopulation of dopamine neurons, allowing for the recruitment of a distinct pool of dopamine neurons. Understanding these mechanisms is essential for elucidating the pathophysiology of various psychiatric disorders and for developing targeted therapeutic interventions.

In this study, we aim to address these knowledge gaps by investigating the role of VP projections in controlling VTA dopamine neuron population activity. We performed in vivo electrophysiology to record from dopamine neurons while simultaneously stimulating the VP to determine the percentage of spontaneously active dopamine neurons that can be inhibited by VP. Furthermore, we utilized a monosynaptic tracer to map the projections from VP to VTA, providing an anatomical framework for understanding these interactions. Our findings shed new light on the neural circuits involved in dopaminergic regulation that are disrupted under conditions such as psychosis.

## Materials and Methods

All experiments were performed in accordance with guidelines outlined in the USPH Guide for the Care and Use of Laboratory animals and were approved by the Institutional Animal Care and the Use Committees of UT Health San Antonio and the US Department of Veterans Affairs.

### Animals

Studies were performed on adult male and female Sprague Dawley rats (250–600 g; Envigo) that were group-housed (2–3 per cage). All rats were housed in a temperature-controlled environment, maintained on a 12 h light/12 h dark cycle, and provided with *ad libitum* access to food and water.

### Dopamine neuron electrophysiology

Rats were anesthetized with 8% chloral hydrate (400 mg/kg, i.p.; C8383, Sigma-Aldrich) and placed in a stereotaxic apparatus for the duration of the experiments. Supplemental anesthesia was administered as required to maintain suppression of the limb compression withdrawal reflex. A thermostatically controlled heating pad (PhysioSuite, Kent Scientific) was used to maintain a core body temperature of 37°C. Extracellular glass microelectrodes (impedance ∼6–10 MΩ) were lowered into the VTA (AP −5.3 mm, ML ±0.6 mm from the bregma, and DV −6.5 to 9.0 mm ventral of the brain surface) using a hydraulic micropositioner (Model 640; Kopf Instruments). Spontaneously active dopamine neurons were identified using previously established electrophysiological criteria (action potential >2 ms, firing rate between 0.5 and 15 Hz; Grace and Bunney, 1983; Ungless and Grace, 2012) and open filter settings (low-frequency cutoff, 30 Hz; high-frequency cutoff, 30 kHz). The electrodes were lowered to make 6–12 vertical passes through the VTA in a predetermined pattern separated by 200 µm, thereby allowing various regions of the VTA to be sampled. Three parameters of dopamine activity were measured and analyzed: the number of dopamine neurons firing spontaneously (population activity; Grace and Bunney, 1983), basal firing rate, and proportion of action potentials occurring in bursts (defined as the incidence of spikes with <80 ms between them; termination of the burst is defined as >160 ms between spikes; Grace and Bunney, 1983). Analysis of dopamine neuron activity was performed using commercially available computer software (LabChart version 8; ADInstruments).

**Figure 2.**
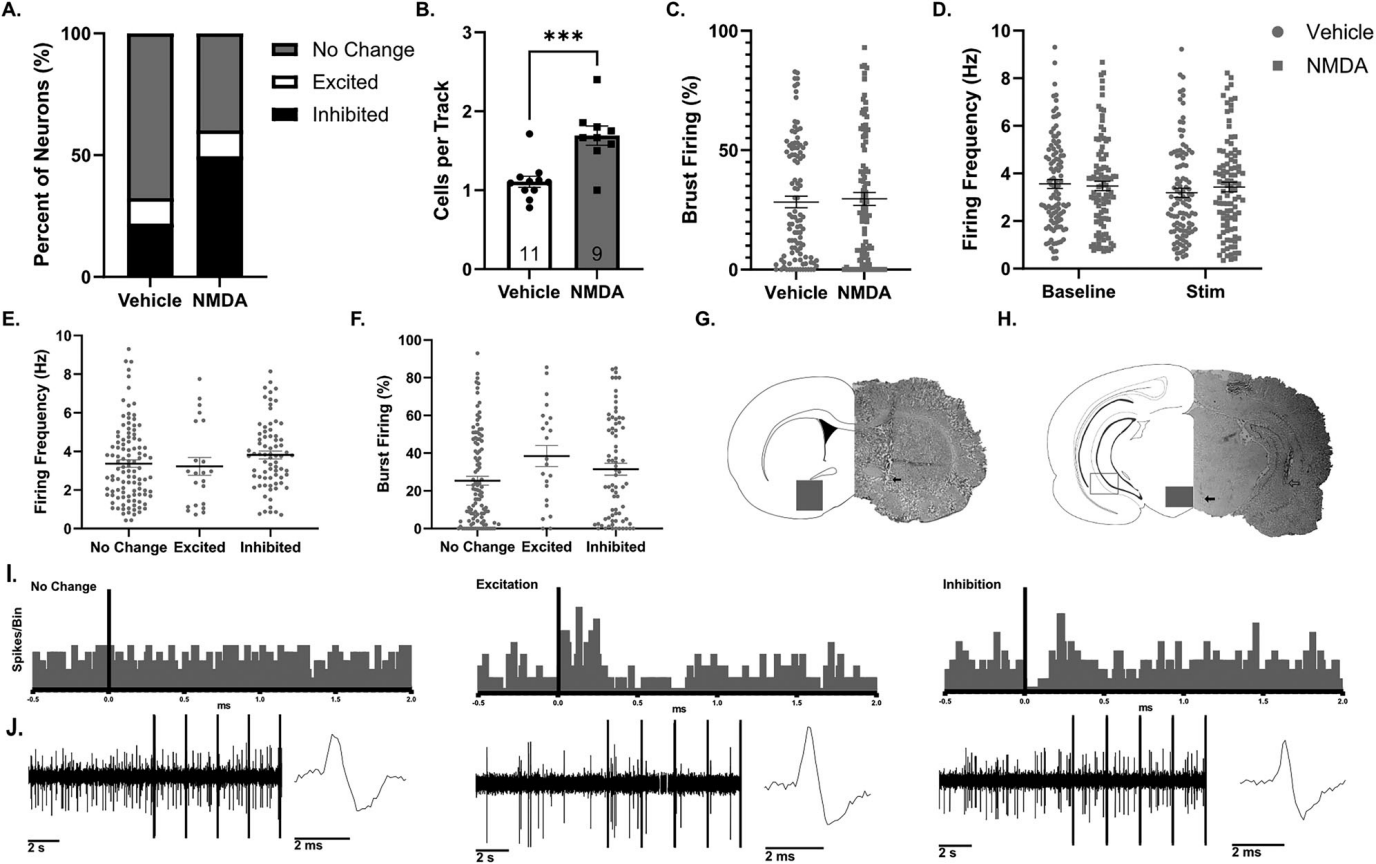
The proportion of VTA dopamine neurons inhibited by VP stimulation increases after vHipp activation. ***A***, The proportion of VTA dopamine neurons unaffected, activated, or inhibited by VP stimulation after delivery of vehicle or NMDA into the vHipp. ***B***, The number of spontaneously active VTA dopamine neurons (population activity) after delivery of vehicle or NMDA into the vHipp. ***C***, The percentage of action potentials firing in bursts. ***D***, Firing frequency of recorded dopamine neurons at the baseline and during VP stimulation. ***E***, ***F***, Average firing frequency (***E***) or percentage action potentials firing in bursts (***F***) of dopamine neurons unaffected, excited, or inhibited by VP stimulation. ***G***, ***H***, Representative brain slices of (***G***) VP (gray square) with stimulating electrode placement (arrow), (***H***) vHipp (open square) with cannula placement (open arrow), and VTA (gray square) with recording electrode placement (black arrow). ***I***, Representative peristimulus time histograms for dopamine neurons unaffected, excited, or inhibited by VP stimulation. ***J***, Representative electrophysiology recording of dopamine neurons before and during VP stimulations (black bar) and action potential traces for dopamine neuron unaffected, excited, or inhibited by VP stimulation. ***p < 0.001.

To examine VP regulation of the VTA, a bipolar, concentric-stimulating electrode (World Precision Instruments) was lowered into the VP (AP −0.0 mm, ML ±2.3 mm from the bregma, and DV −9.0 mm ventral of the brain surface). Once a stable spontaneously active dopamine neuron was identified, a baseline of 1 min was recorded followed by 2 min of VP stimulation using single-current pulses (0.25 ms; 1.0 mA; 0.5 Hz; Fig. 2*J*). To determine whether a dopamine neuron was excited or inhibited by the electrical stimulation, peristimulus time histograms (Fig. 2*I*) were generated and criteria of ≥2 consecutive bins (50 ms) that were greater (for excited neurons) or lower (for inhibited neurons) than two standard deviations from the average baseline (Lodge, 2011). For neurons where two standard deviations below the baseline was negative, a criteria of ≥2 consecutive bins of 0 was used to define inhibition.

For intracranial drug administration of NMDA (0.75 µl of 1.5 mg/ml; M3262, Sigma-Aldrich) or vehicle [Dulbecco's PBS; 0.75 µl; 59331C, Sigma-Aldrich], a 26 gauge cannula (Plastics One) was lowered into the vHipp (AP −5.3 mm, ML ± 5.0 mm from the bregma, DV −7.0 mm ventral of the brain surface). An internal cannula (Plastics One), extending 1 mm past the end of the guide cannula was used to deliver a one-time injection administered at a rate of 0.5 µl/min ∼10 min prior to electrophysiology recordings.

### Trans-synaptic viral tracing

Rats were anesthetized with Fluriso (2–5% isoflurane, USP, with oxygen flow at 1 L/min) and placed in a stereotaxic apparatus using blunt atraumatic ear bars. A core body temperature of 37°C was maintained. Rats had a cannula implanted bilaterally in the VP (AP −0.0 mm, ML±2.3 mm from the bregma, and DV −8.0 mm ventral of the brain surface), and the Herpes helper virus AAV2-hSYN-TK was injected (Zeng et al., 2017; 0.5 µl/2 × 10^11^ GC/ml; VectorBuilder) through an injector that extended 1 mm past the cannula tip. Three weeks following the initial injection, the replication-incompetent monosynaptic anterograde Herpes simplex virus Type 1 strain 129 (H129-ΔTK-tdTomato) was bilaterally injected in the VP (Zeng et al., 2017; UCI; 0.5 μl/2.75 × 10^5^ PFU). This virus has a deleted thymidine kinase (TK) gene stopping it from replicating or spreading in neurons. When complementarily expressed with TK from the helper virus, H129-ΔTK-tdT can map the direct monosynaptic projections of the transfected neurons (Zeng et al., 2017).

### Immunohistochemistry

Ten days after the H129-ΔTK-tdTomato administration, rats were deeply anesthetized with 8% chloral hydrate (400 mg/kg, i.p.) and transcardially perfused with saline followed by 4% formaldehyde. Brains were removed, postfixed overnight, and cryoprotected (10% w/v sucrose in PBS) until saturated. Brains were sectioned coronally (50 µm) using a cryostat (Leica Biosystems), and sections containing the VTA were stored in PBS. Sections were washed three times (10 min) in PBS then blocked (2% normal goat serum, 0.3% Triton X-100) for 30 min at room temperature. Primary antibodies [rabbit anti-tyrosine hydroxylase 1:1,000 (ab112, Abcam); chicken anti-red fluorescent protein 1:1,000 (409 006, Synaptic Systems)] were applied (in PBS containing 1% normal goat serum, 0.3% Triton X-100) overnight at 4°C. Secondary antibodies [Alexa Fluor 488 goat anti-rabbit 1:1,000 (A-11008, Thermo Fisher Scientific); Alexa Fluor 594 goat anti-chicken 1:1,000 (A-11042, Thermo Fisher)] for 1 h at room temperature. Slices were then mounted and coverslipped with ProLong Gold antifade reagent (P36930, Thermo Fisher Scientific).

### Monosynaptic neuron quantification

Immunostained sections (2–5 per animal) were imaged using an inverted fluorescent microscope (Axio Observer Z1, Carl Zeiss) with 63× oil objective to capture high-resolution images of VTA. Cell quantification was performed manually using the ImageJ software with the Colocalization Object Counter (Lunde et al., 2020). Dopamine neurons were quantified by counting TH+ cells in the green 488 channel, while cells receiving direct monosynaptic input from the VP were quantified by counting tdTomato+ cells in the red 594 channel. To determine the spatial distribution of TH+ and tdTomato+ cells, sections were grouped and analyzed according to stereotaxic coordinates. For each slice, the VTA was divided into three sections from medial to lateral ([0.00–0.60], [0.60–1.3], [1.3–2.0] mm from the bregma). Brain sections were categorized into four groups from anterior to posterior ([−4.7 to −5.0], [−5.0 to −5.4], [−5.4 to −5.8], and [−5.8 to −6.1] mm from the bregma).

### Histological verification of electrode locations

**Figure 3.**
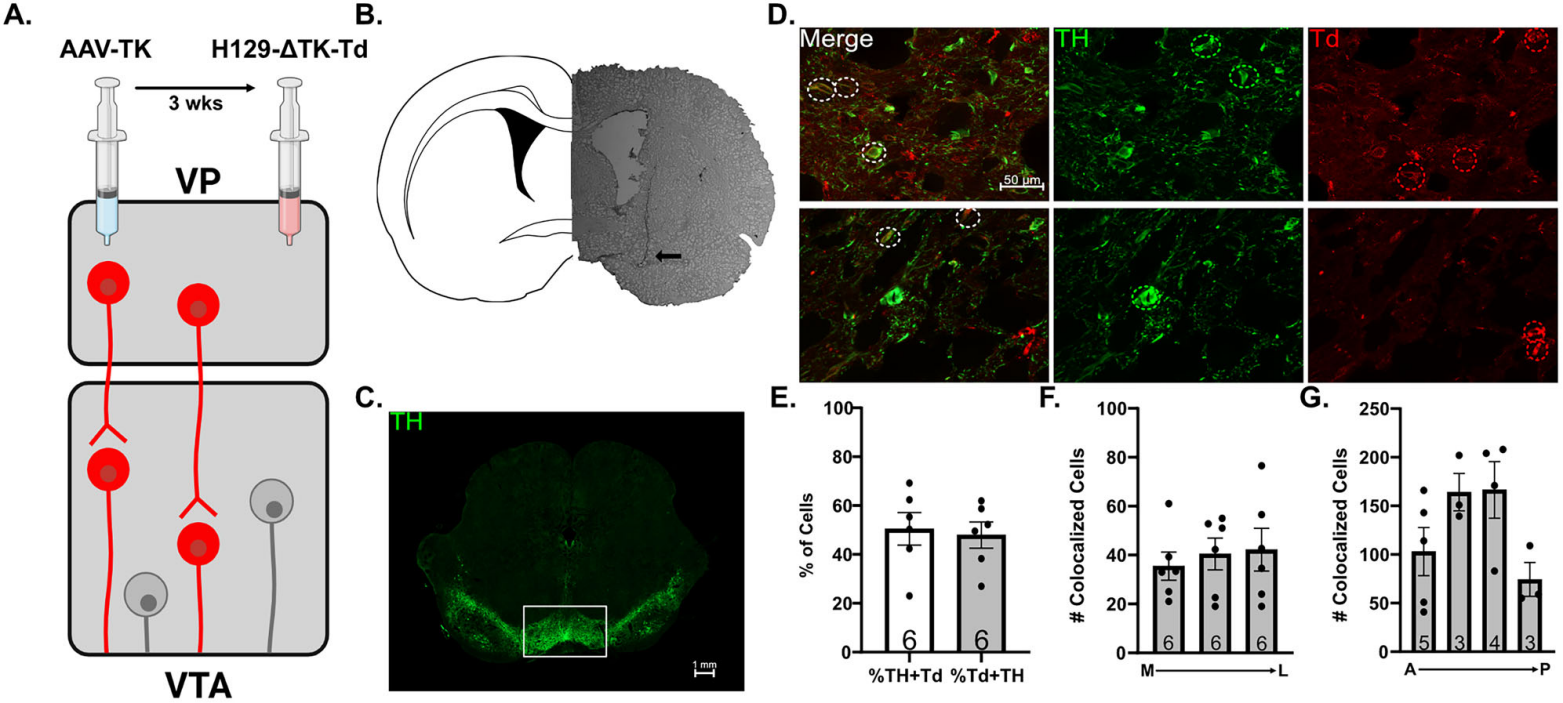
The VP projects to a subset of VTA dopamine neurons. ***A***, Schematic depicting monosynaptic labeling using H129 and helper virus and injection timeline. ***B***, Representative brain slice of VP with cannula placement (arrow). ***C***, Representative brain slice of mesencephalon containing VTA and tyrosine hydroxylase (TH) immunolabeling (green). ***D***, Representative images of cells in VTA indicating colocalized cells (left, white circles), TH+ only cells (middle, green circles), and tdTomato (Td)+ only cells (right, red circles). ***E***, The percentage of TH+ cells colabeled with Td (left) and the percentage of Td+ cells colabeled with TH (right). ***F***, The number of colocalized cells along the medial (M)/lateral (L) axis. ***G***, The number of colocalized cells along the anterior (A)/posterior (P) axis.

Rats used for electrophysiology were rapidly decapitated following recordings, and brains were dissected out, postfixed, cryoprotected, and sectioned coronally (25 µm). Sections containing electrode or cannula tracks were mounted onto gelatin–chrome-coated slides, stained with neutral red (0.1%) and thionin acetate (0.01%), and coverslipped with DPX Mountant (06522, Sigma-Aldrich) for histological confirmation of the recording electrode (Fig. 2*G*), stimulating electrode (Fig. 2*G*), or cannula (vHipp, Fig. 2*H*; VP, Fig. 3*B*) with reference to a stereotaxic atlas (Paxinos and Watson, 1998).

### Statistical analysis

Electrophysiological analysis of dopamine neuron activity was performed using the commercially available computer software (Lab Chart version 8; ADInstruments). Data are represented as the mean ± SEM with *n* values representing the number of rats per experimental group, unless otherwise stated. Proportions of dopamine neurons inhibited and unchanged by VP stimulation were determined by *χ*^2^, while other metrics of dopamine neuron activity were analyzed by *t* test or two-way ANOVA (vehicle/NMDA × stimulation on/off) and the Holm–Sidak post hoc test was used when significant interactions were determined (Table 1). When assumptions of normality were violated, Mann–Whitney or Aligned Rank Transform (ART) ANOVA was used. Statistics were calculated using the Prism software (Version 10; GraphPad Software) or R-4.5.0 with significance determined at *p* < 0.05 and graphed with the Prism software.

**Table 1.** Statistical table indicating data structure and confidence intervals for all statistical analysis

	Figure	Data structure	Type of test	Power
a	Figure 2*A*	Contingency table	*χ* ^2^	N/A
b	Figure 2*B*	Normal distribution	*t* test	95% CI [0.31,0.88]
c	Figure 2*C*	Not normally distributed	Mann–Whitney	95% CI [−5.0,6.2]
d	Figure 2*D*	Not normally distributed	ART ANOVA	95% CI Veh [188,221] NMDA [194,227] BL [198,231] Stim [184,217]
e	Figure 2*E*	Not normally distributed	Kruskal–Wallis	95% CI [2.98,3.72; 2.27,4.19; 3.41,4.23]
f	Figure 2*F*	Not normally distributed	Kruskal–Wallis	95% CI [20.92,30.05; 26.78,50.17; 25.25,37.75]
g	Figure 3*E*	Normal distribution	*t* test	95% CI [−21.6 to 16.6]
h	Figure 3*F*	Normal distribution	One-way ANOVA	95% CI [20.64,50.27; 23.81,57.08; 19.66,64.76]
i	Figure 3*G*	Normal distribution	One-way ANOVA	95% CI [34.32,171.7; 105.4,210.6; 74.08,258.9; −0.4113,149.1]

## Results

### Proportion of dopamine neurons inhibited by VP stimulation increases following vHipp activation

To determine the proportion of VTA dopamine neurons inhibited by VP stimulation under normal conditions and during hippocampal activation, we used in vivo extracellular electrophysiology with electrical stimulation of the VP (Fig. 2). In vehicle-treated animals, 21.9% of dopamine neurons were inhibited by VP stimulation, 10.5% were excited, and 67.6% exhibited no change (*n* = 105 dopamine neurons from 11 rats; Fig. 2*A*). We next wanted to determine if activation of the vHipp, which indirectly attenuates inhibition from the VP to the VTA, would change the proportion of dopamine neurons responding to VP stimulation. Interestingly, following NMDA activation of vHipp, the percentage of dopamine neurons inhibited by VP stimulation increased to 49.5% and the corresponding dopamine neurons that did not respond to stimulation decreased to 39.8% (*n* = 103 dopamine neurons, nine rats). The percentage of excited neurons remained the same at 10.7%. There was a significant difference in the proportion of inhibited and unchanged neurons between vehicle and NMDA-treated animals (*χ*^2^ = 18.61; *p* < 0.0001; Fig. 2*A*).

Consistent with previous studies, rats receiving vehicle in vHipp exhibited 1.11 ± 0.07 cells per track (*n* = 11 rats), and this was significantly increased to 1.70 ± 0.12 cells per track (*n* = 9 rats) following NMDA administration (*t* test; *t* = 4.38; *p* = 0.0004; Fig. 2*B*). Furthermore, vHipp activation did not alter burst firing (*n* = 99 neurons, 29.6 ± 2.7% burst firing) compared with vehicle (*n* = 105 neurons, 28.3 ± 2.4% burst firing; Mann–Whitney; *U* = 5,131; *p* = 0.875; Fig. 2*C*). vHipp NMDA also did not alter the baseline firing rate (*n* = 99 neurons, 3.47 ± 0.19 Hz) compared with vehicle (*n* = 105 neurons; 3.56 ± 0.19 Hz; *t* test; *t* = 0.361; *p* = 0.719). Repeated VP stimulation had no sustained effect on the average firing rate in vehicle (*n* = 105 neurons; 3.19 ± 0.19 Hz) or NMDA (*n* = 99 neurons; 3.43 ± 0.2 Hz; ART ANOVA; *F*_(1,407)Stimulation_ = 1.287; *p* = 0.257; *F*_(1,407)Treatment_ = 0.282; *p* = 0.596; Fig. 2*D*) treated animals. Additionally, we examined whether firing frequency or burst firing predict the response to VP stimulation. Interestingly we found no effect of stimulation response on firing frequency (no change, *n* = 108 neurons; 3.37 ± 0.19 Hz; excited, *n* = 22 neurons; 3.23 ± 0.46 Hz; inhibited, *n* = 74 neurons; 3.82 ± 0.21 Hz; Kruskal–Wallis, KW = 4.622; *p* = 0.0922; Fig. 2*E*) or burst firing (no change, 25.40 ± 2.34%; excited, 38.47 ± 5.62%; inhibited, 31.5 ± 3.14%; Kruskal–Wallis, KW = 5.691; *p* = 0.058; Fig. 2*F*). Taken together, these data support the hypothesis that the VP regulates tonic inhibition of VTA dopamine neurons. Given that the proportion of dopamine neurons inhibited by VP is increased under NMDA activation of vHipp, it is likely that the VP innervates a subset of dopamine neurons in the VTA, rather than regulating a more sizable proportion of the population of neurons.

### Monosynaptic projections from the VP innervate ∼50% of VTA dopamine neurons

To validate these findings anatomically, we used the anterograde monosynaptic Herpes virus to label projections from the VP to VTA (Fig. 3*A*). We used a TH antibody to label dopamine neurons in the VTA (Fig. 3*C*) and counted the proportion of dopamine neurons receiving monosynaptic inputs from the VP. We found that 50.4 ± 6.7% of TH+ cells were tdTomato+, indicating that VP innervates about half of dopamine neurons in VTA (Fig. 3*D*,*E*). Interestingly, 47.9 ± 5.4% of tdTomato+ cells were TH+, indicating that VP also innervates a substantial number of nondopaminergic cells in VTA. To determine if dopamine neurons innervated by the VP were localized to specific regions of VTA, we analyzed colocalized cells based on anatomical position. The average number of colocalized cells per animal was 35.5 ± 5.8 for the most medial portion of VTA, 40.5 ± 6.5 in the center, and 42.2 ± 8.8 for the most lateral portion of VTA. One-way ANOVA revealed no significant differences in colocalized cells between VP subregions medial to lateral (*F*_(2,15)_ = 0.2423; *p* = 0.7879; Fig. 3*F*). Similarly, no significant differences were observed between subregions anterior to posterior [(anterior→posterior) 103.0 ± 24.7; 164.2 ± 19.2; 166.5 ± 29.0; 74.3 ± 17.4; *F*_(3,11) _= 2.999; *p* = 0.0769; Fig. 3*G*]. These data indicate that VP heterogeneously innervates a subset of dopamine neurons in the VTA.

## Discussion

Aberrant dopamine system function contributes to numerous psychiatric disorders including psychosis, which is believed to be driven by an increase in the number of spontaneously active dopamine neurons in the VTA (Abi-Dargham, 2004; Lodge and Grace, 2008b; Howes et al., 2009; Perez et al., 2022). This activity state, termed population activity, has been extensively examined and known to be regulated by multisynaptic circuits involving many brain regions, including the PVT, BLA, vHipp, and NAc, ultimately converging on VP inputs to the VTA (Lodge and Grace, 2008b; Chang and Grace, 2013a,b; Perez and Lodge, 2018). In vivo intracellular recordings from VTA dopamine neurons have demonstrated that a proportion of these neurons are bombarded by miniature inhibitory postsynaptic potentials, likely from the VP, which results in an absence of spontaneous activity (Grace and Bunney, 1985). This activity state seems essential for the proper functioning of the dopamine system, but it remains unclear whether a majority of VTA dopamine neurons are innervated by the VP. Furthermore, it is not clear if this connection orchestrates the activation of specific neurons or if only a particular subset of neurons are inhibited by the VP resulting in a silent “pool” of neurons available to be recruited. Here, we demonstrate that the second theory appears correct: the VP only innervates a subset of VTA dopamine neurons that are distinguishable based on their response to VP stimulation under our recording conditions. This indicates that changes in the overall activity of dopamine neurons are influenced by the activity of a distinct collection of dopamine neurons that are selectively innervated by the VP.

Studies on the innervation from VP to the VTA consistently indicate that VP GABA neurons project to VTA dopamine neurons (Faget et al., 2016; Li et al., 2020; Palmer et al., 2024). Retrograde tracing studies indicate that projections from the VP to VTA are largely inhibitory, as over 90% of VP neurons projecting to the VTA express the GABA synthesizing enzyme glutamic acid decarboxylase (Palmer et al., 2024). However, previous studies have not investigated the specific VTA neuron population receiving input from the VP. In our studies, we employ extracellular electrophysiology to record from individual dopamine neurons in vivo. We found that at the baseline, ∼25% of VTA dopamine neurons were inhibited by VP stimulation, suggesting that the majority of spontaneously active dopamine neurons examined under control conditions are not directly innervated by the VP. This further suggests that a subset of dopamine neurons (∼25%) receive input from the VP that is not tonically active and may be recruited to decrease dopamine neuron population activity. It is important to note that other parameters of the dopamine neuron recorded such as the firing rate and burst firing did not correlate with response to VP stimulation, suggesting that VP activity regulates population activity specifically. Indeed, previous studies have demonstrated that chronic mild stress produces significant decreases in dopamine neuron population activity that are dependent on an increased VP drive from the BLA that may be associated with symptoms of major depressive disorder (Chang and Grace, 2013a). It should be noted that previous studies have not reported decreased dopamine neuron population activity following activation of the VP (Floresco et al., 2003); however, these studies used bicuculline, a GABA_A_ receptor antagonist, suggesting that the VP inputs to the VTA may not be tonically inhibited but can be activated by glutamatergic afferents to modulate dopamine neuron activity. Furthermore, a small population of VTA dopamine neurons (∼10%) was activated by VP stimulation. Several reasons could underlie this observation. The first is that, the VP innervates a number of nondopaminergic neurons in the VTA (Faget et al., 2016). VP inhibition of VTA GABA interneurons would, therefore, result in the activation of a small proportion of dopamine neurons. The other potential rationale is that glutamate may be coreleased by a subset of VP neurons, as previous studies have reported that ∼25% of VP projections to the VTA are positive for VGlut2, a vesicular glutamate transporter (Palmer et al., 2024). Our anatomical data indicate that roughly half of VP-targeted VTA neurons are not TH-positive, consistent with projections to local GABAergic and glutamatergic neurons. These nondopaminergic populations may contribute to VP influence over VTA output by providing feedforward inhibition or excitation of dopamine neurons or by engaging downstream targets directly. Although not examined functionally here, their potential roles warrant further investigation.

The current literature posits that a tonic drive from the VP results in the inhibition of a significant population of VTA dopamine neurons under control conditions (Floresco et al., 2003; Aguilar et al., 2014; Root et al., 2015; Palmer et al., 2024). Therefore, it is impractical to examine whether these dopamine neurons are inhibited by VP stimulation if they are not spontaneously active. To address this caveat, we activated the vHipp, which is known to reduce VP drive to the VTA and increase the number of spontaneously active dopamine neurons (Perez and Lodge, 2018). It is important to note that our observed increase in dopamine neuron population activity was slightly lower than what has been reported in previous studies (Lodge and Grace, 2006), and this may raise the question of whether all VP-inhibited dopamine neurons were fully disinhibited. It is possible that repeated VP stimulation may lead to slight suppression in the number of spontaneously active dopamine neurons, suggesting that our reported number of inhibited neurons may be an underestimation of the actual number. Nevertheless, we found that the number of dopamine neurons inhibited by VP stimulation drastically increased. These findings suggest that a significant proportion of VTA dopamine neurons (∼50%) receive input from the VP and that under control conditions, these inputs provide a tonic inhibition over a subpopulation of VTA dopamine neurons.

To confirm our physiological findings, we used a monosynaptic tracer to anatomically map the projections from the VP to the VTA. We found that about half of the dopamine neurons in the VTA received monosynaptic innervation from VP, consistent with data from our electrophysiology experiments. Interestingly, a significant number of nondopamine cells were also innervated by the VP. The VTA is comprised of ∼60% dopamine neurons, ∼35% GABA neurons, and only ∼5% glutamate neurons; thus, these other cells innervated by the VP are likely predominantly GABAergic (Nair-Roberts et al., 2008). These nondopaminergic populations may contribute to VP influence over VTA output by providing feedforward inhibition or excitation of dopamine neurons or by engaging downstream targets directly. Although not examined functionally here, this may correlate with our electrophysiological findings that a small proportion of VTA dopamine neurons are excited by VP stimulation, as inhibition of GABAergic interneurons may lead to excitation of dopamine neurons. These potential roles warrant further investigation. While the VP regulation of dopamine neurons in the VTA has been implicated in a variety of pathological conditions (Sonnenschein et al., 2020; Soares-Cunha and Heinsbroek, 2023), the consequence of VP regulation of GABAergic cells is less well studied and requires further elucidation.

One caveat of our studies is the limitations of H129-derived anterograde tracers. H129-dTK vectors may fail to yield high labeling intensity due to the limited fluorescent protein level (Zeng et al., 2017); thus, we amplified the signal using immunohistochemistry. However, the occurrence of incomplete labeling may have led to an underestimation of the total number of VP-innervated neurons in the VTA. Additionally, viral tropism could introduce cell-type biases that favor transfection of certain neuronal subpopulations. Finally, despite H129 primarily labeling in an anterograde manner, H129 is occasionally transported retrogradely from terminals in the injected regions. As a consequence, we cannot fully rule out the possibility that some labeled cells are VP-projecting neurons from VTA. These caveats suggest that our estimate of ∼50% VP innervation should be interpreted as an approximation rather than an absolute measure of connectivity.

Previous studies have identified subregion-specific projection patterns from the VP to the VTA (Zahm et al., 2011; Root et al., 2015; Soares-Cunha and Heinsbroek, 2023). The VP is divided into distinct subregions based on functional anatomy and projection targets, with the ventromedial subregion exhibiting the strongest projections to the VTA (Root et al., 2015; Soares-Cunha and Heinsbroek, 2023). Based on this evidence, our coordinates for VP were selected to focus on this ventromedial region. To investigate whether the VP projections to the VTA resided in a specific subregion, we evaluated the distribution of dopamine neurons innervated by VP across the medial/later and anterior/posterior axes of the VTA. Comparisons of colocalized cells along these axes of the VTA revealed no significant differences, suggesting that VP afferents are evenly distributed throughout the VTA. Consistent with this finding, anterograde tracing with *Phaseolus vulgaris*–leucoagglutinin injected into the VP resulted in robust labeling of the VTA without evidence of spatially distinct projection patterns (Zahm et al., 2011). These findings suggest that the VP→VTA pathway operates in a spatially heterogeneous manner, allowing the VP to exert widespread influence over dopamine neuron activity across the VTA. Another important consideration is the cellular heterogeneity within VP itself. The VP contains multiple projection neuron classes, including purely GABAergic neurons, purely glutamatergic neurons, and a population that coreleases GABA and glutamate (Root et al., 2018). This observation indicates that at least a part of the VP input to DA neurons may be excitatory or modulatory rather than purely inhibitory. Such heterogeneity likely contributes to the diverse functional outcomes observed in our recordings, where VP stimulation produces inhibition in some dopamine units but had little effect, or even promoted firing, in others. Moreover, as discussed above, the observation that the VP projects to nondopaminergic neurons further confounds the interpretation where multisynaptic increases and decreases in dopamine neuron firing may be observed. Corelease adds an additional layer of complexity, as glutamate transmission can depolarize dopamine neurons, making them more susceptible to shunting inhibition from concurrent GABA_A_ receptor activation, or alternatively drive rebound firing following GABAergic inhibition. Integrating these findings with the known diversity of VP outputs supports a model in which VP exerts a nuanced, projection- and state-dependent control over dopamine neuron population activity rather than acting as a purely inhibitory gate.

The apparent discrepancy between the proportion of dopamine neurons anatomically innervated by VP (∼50%) and those functionally inhibited under baseline conditions (∼25%) may partly reflect the tonic nature of VP output. VP neurons are known to fire tonically at rest (Clark, 2020) and provide a persistent GABAergic tone to VTA (Floresco et al., 2001, 2003; Sesack and Grace, 2009). This tonic input likely holds a substantial fraction of dopamine neurons below firing threshold, effectively rendering them “silent” in our population sample. Consequently, these neurons cannot be detected as inhibited by additional VP stimulation because they are already suppressed. vHipp activation, by engaging the NAc→VP pathway, reduces VP activity and disinhibits these quiescent DA neurons, increasing population activity. Once these neurons enter the spiking pool, VP stimulation can reveal their latent sensitivity, resulting in the larger proportion (>50%) of inhibited DA neurons observed after vHipp drive which is consistent with the anatomical data.

Beyond tonic inhibition and state-dependent sampling, other mechanisms may also contribute to this apparent disparity in control animals. VP terminals likely exhibit variability in synaptic strength and release probability, such that weaker inputs may only exert detectable effects when dopamine neurons are depolarized (although we did not see a correlation between baseline firing frequency and the effect of VP stimulation). Some VP terminals could represent functionally quiescent synapses lacking postsynaptic GABA_A_ receptors or expressing primarily GABA_B_ receptors that produce slower, subthreshold inhibition (see if there are references to support this). Postsynaptic heterogeneity is also well described among dopamine neuron subtypes, with differences in chloride driving force, GABA receptor expression, and intrinsic excitability that may shape their responsiveness to VP input (Ford et al., 2006; Lammel et al., 2008). Finally, VP projections to VTA are phenotypically diverse, with both GABAergic and glutamatergic terminals (and possible corelease), and target both VTA GABA and dopamine neurons (Soares-Cunha and Heinsbroek, 2023). Together, these factors provide a mechanistic framework that reconciles our anatomical tracing data with the observed functional inhibition and underscores the dynamic, state-dependent influence of VP on dopamine population activity.

Dopamine neuron population activity has gained increasing attention over the last 20 years, as it is thought to provide a gain of function to the dopamine system, allowing for modulation of the number of spontaneously active dopamine neurons available to respond to behaviorally relevant stimuli (Grace, 2016). This form of regulation is essential for assigning salience to environmental cues and ensuring appropriate behavioral responses (Berridge and Robinson, 1998). Disruptions in this process have been implicated in a range of neuropsychiatric disorders. For example, decreases in dopamine neuron population activity following chronic stress have been associated with the pathophysiology of depression, leading to blunted dopaminergic responses and despair-like phenotypes dependent on actions through the BLA (Chang and Grace, 2013a,b). Conversely, rodent modes used to study schizophrenia, such as the methylazoxymethanol acetate model, show a significant increase in dopamine neuron population activity, via hippocampal hyperactivity, which contributes to aberrant salience attribution and the emergence of positive symptoms (Lodge and Grace, 2007, 2008b; Lodge, 2013). Moreover, acute stressors such as footshock have also been shown to increase dopamine neuron population activity, indicating that stress can profoundly modulate dopaminergic tone chronically and acutely (Elam et al., 2021; McCoy et al., 2022). Similar enhancements in dopamine system function have been observed in the context of substance abuse, where repeated administration of psychostimulant drugs like amphetamine increase dopamine neuron population activity leading to behavioral sensitization (Lodge and Grace, 2008a). Taken together, understanding of the afferent regulation of dopamine neuron population activity is of central importance. Under physiological conditions, such input may help fine-tune salience or reward processing by calibrating the dopaminergic gain in response to internal states or external cues. However, in pathological states, alterations in VP function could lead to maladaptive dopamine system output, contributing to the behavioral and cognitive impairments observed across a range of psychiatric disorders. Thus, delineating the various circuitries that regulate dopamine neuron population activity, particularly under conditions that model psychiatric illness, provides a valuable framework for identifying potential therapeutic targets aimed at restoring dopamine system function.

Here we provide important information examining the mechanisms underlying this critical regulator of dopamine system function that is essential for our understanding of both normal salience function and a number of psychiatric disorders. Specifically, the observation that the VP does not innervate all dopamine neurons in the VTA suggests that innervated neurons may belong to distinct groups, likely characterized by specific molecular phenotypes and potentially projection patterns. Indeed, dopamine neurons project to a wide variety of cortical and subcortical regions including the medial prefrontal cortex, ventral striatum, and amygdala (Haber et al., 1985). Studies by Floresco and Grace have demonstrated increases in tonic dopamine efflux in the NAc following VP inactivation suggesting that the VTA–NAc neurons may be preferentially innervated by the VP (Floresco et al., 2003); however, this requires further elucidation. Future studies will focus on classifying the precise phenotypes of both dopamine and nondopamine neurons in the VTA that receive innervation from the VP as well as their specific projection targets. Taken together, these findings have important implications for better understanding the neuropathology underlying various psychiatric disorders, informing the development of targeted therapeutic interventions.

## Data Availability

All data supporting this article will be shared upon reasonable request to the corresponding author.

## Synthesis

Reviewing Editor: Jibran Khokhar, Western University Department of Anatomy and Cell Biology Decisions are customarily a result of the Reviewing Editor and the peer reviewers coming together and discussing their recommendations until a consensus is reached. When revisions are invited, a fact-based synthesis statement explaining their decision and outlining what is needed to prepare a revision will be listed below. The following reviewer(s) agreed to reveal their identity: Anthony Grace.

This manuscript provides a significant and well-executed contribution to our understanding of how the ventral pallidum (VP) selectively modulates dopamine (DA) neuron activity in the ventral tegmental area (VTA). Using a combination of in vivo electrophysiology and anatomical tracing, the authors demonstrate that VP inputs inhibit or excite only a subset of DA neurons, challenging the classical view of uniform tonic inhibition. The study is methodologically rigorous, and its insights have important implications for our understanding of motivational circuitry and psychiatric dysfunction. However, several methodological and interpretative aspects warrant clarification and deeper discussion to fully support the claims made.

While the study frequently refers to functionally distinct DA neuron populations, these distinctions are not well defined beyond stimulation parameters. It might be beneficial to classify neurons based on combinations of these features (high firing/high bursting vs. low firing/low bursting), which might help better define the functionally distinct populations they refer to.

Anatomical data indicate that about half of VP-targeted VTA neurons are not TH-positive, suggesting a significant input to non-dopaminergic cells, likely GABAergic or glutamatergic. These cells are only briefly acknowledged, yet may contribute meaningfully to circuit function. Including data on these neurons, if available, or at least expanding the discussion on their possible roles would substantially enrich the interpretation.

There is a notable gap between the number of DA neurons anatomically innervated by the VP and those functionally inhibited under baseline conditions. Although the authors attempt to reconcile this discrepancy by showing that ventral hippocampal activation reveals additional VP-mediated inhibition, other mechanisms should be discussed. Possibilities such as variability in synaptic strength, presence of silent synapses, differences in receptor expression across DA subtypes, or heterogeneity in the VP terminal composition (e.g., GABAergic vs. glutamatergic) should be considered.

The manuscript briefly notes that a portion of VP→VTA projections are VGlut2-positive, indicating potential glutamate co-release, but does not fully integrate this observation with broader findings about VP neuronal diversity. Prior work has shown that the VP contains distinct populations-purely GABAergic, purely glutamatergic, and co-releasing neurons. A more detailed discussion of how this diversity might influence DA neuron modulation would strengthen the interpretive framework.

The observed 50% increase in DA neuron activity following vHipp activation is lower than what has been reported in previous studies, which describe closer to a doubling. This discrepancy raises the possibility that not all VP-inhibited neurons were fully disinhibited. A better justification for this level of activation, or a more direct acknowledgment of this caveat, would enhance confidence in the functional interpretation.

The authors use a 3 ms binning threshold to detect changes in spike activity, referencing Lodge et al. (2011), who used a 2 ms threshold. Given that even small differences in bin size can influence classification of responsive neurons, a brief rationale for this methodological choice would clarify its impact on the reported results.

While HSV-based viral tracing is a valuable tool, it is subject to limitations, including incomplete labeling and potential cell-type biases. These caveats are not currently addressed in the manuscript. Even a brief mention of the constraints associated with this method would improve transparency.

The terms "functionally distinct" and "specific subset" are used throughout the manuscript but could benefit from clearer definition. As currently presented, these terms imply intrinsic biological distinctions that are not fully supported by the data.

The anatomical and physiological findings are presented in parallel, but the connection between them would benefit from additional clarity. Presenting quantitative summaries or comparisons, perhaps in a figure or table, would help clarify the degree of overlap between traced and functionally modulated neurons.
